# Psychopharmacological Treatment and Psychological Interventions in Irritable Bowel Syndrome

**DOI:** 10.1155/2012/486067

**Published:** 2012-08-22

**Authors:** Emanuele Sinagra, Claudia Romano, Mario Cottone

**Affiliations:** Division of Internal Medicine “Villa Sofia-V. Cervello” Hospital, University of Palermo, Via Trabucco 180, 90146 Palermo, Italy

## Abstract

Irritable bowel syndrome (IBS) accounts for 25% of gastroenterology output practice, making it one of the most common disorders in this practice. Psychological and social factors may affect the development of this chronic disorder. Furthermore, psychiatric symptoms and psychiatric diseases are highly prevalent in this condition, but the approach to treating these is not always straightforward. As emphasized in the biopsychosocial model of IBS, with regard to the modulatory role of stress-related brain-gut interactions and association of the disease with psychological factors and emotional state, it proves useful to encourage psychopharmacological treatments and psychosocial therapies, both aiming at reducing stress perception. The aim of this paper is to analyze the effectiveness of psychopharmacological treatment and psychological interventions on irritable bowel syndrome.

## 1. Introduction

Irritable bowel syndrome (IBS) is a chronic, relapsing, and remitting functional disorder of the gastrointestinal (GI) tract for which there is no known structural or anatomical explanation.

Its prevalence in the general population is estimated to be between 5% and 20% [1–4], accounting for up to 25% of gastroenterology output practice [5]. The presence of IBS is defined by clinical criteria, which include the presence of abdominal pain, or discomfort, and alterations in bowel habits, in the absence of red flag alarm features, such as weight loss or anemia [6].

IBS is defined by the Rome III criteria as “symptoms of recurrent abdominal pain or discomfort and a marked change in bowel habits for at least six months, with symptoms experienced on at least three days of at least three months, with two of the three following findings: (a) pain is relieved by a bowel movement; (b) onset of pain is related to a change in frequency of stool; (c) onset of pain is related to a change in appearance of stool” [7].

The cause of IBS is actually unknown, but probably it is unlikely that a single factor is responsible for the diverse presentations of this heterogeneous and complex disorder. In fact, IBS has a multifactorial etiology, involving altered gut reactivity and motility, altered pain perception, and alteration of the brain-gut axis [8]. In addition, psychological and social factors can influence digestive function, symptom perception, illness behavior, and outcome [9]. According to the biopsychosocial model of IBS, symptoms are both determined and modified by psychological and social influences, and the link between psychosocial factors and GI functions is through the brain-gut axis [10, 11].

The brain-gut axis allows bidirectional input and thus links emotional and cognitive centers of the brain with peripheral functioning of the GI tract and vice versa. Hence extrinsic (vision, smell, etc.) or enteroceptive (emotion, thought) information has, by nature of its neural connections from higher centers, the capacity to affect GI sensation, motility, secretion, and inflammation. Conversely, viscerotropic effects (e.g., visceral afferent communications to the brain) reciprocally affect central pain perception, mood, and behavior [12].

Since the biopsychosocial model of IBS was developed, there has been constantly growing interest in the influence of psychosocial factors on the pathogenesis and clinical course of IBS [8].

Psychological and social factors may already affect the development of IBS early in life, conditioning one's psychosocial development, and during life, leading to gut dysfunction and dysregulation of the brain-gut axis, through the alteration of digestive functions (motility, sensation, inflammation), symptom perception, and illness behavior [11].

Studies about IBS clustering in families show that environmental factors may play a role, together with inherited mechanisms, in the development of IBS [13, 14]. A history of abuse represents a particularly important factor leading to increased psychological distress [15–21].

Personality traits are also implicated in the pathogenesis of IBS and in the decision to seek medical help [8]. Neuroticism (considered as the tendency to experience negative emotions) and alexithymia (defined as difficulty in identifying feelings and distinguishing between feelings and bodily sensations) are the most prevalent traits; furthermore, neuroticism is a predictor of illness perception and influences coping strategies [22–25].

Furthermore, patients with IBS often present irrational health beliefs, leading to hypochondriac attitudes and respond to their illness adopting different coping strategies, compared with patients with organic diseases or healthy controls [26–28].

Finally, psychiatric symptoms and psychiatric diseases are frequent in IBS, especially in severe forms. Conversely, patients with severe IBS may have more than one psychiatric disorder [29–32]. Particularly, depression is the most common psychiatric disorder in IBS, involving approximately 30% of patients. In this subset of patients, high levels of somatization determine frequent use of health care services, poor response to treatment and poor health-related quality of life [28, 33–38].

As emphasized in the biopsychosocial model of IBS, with regard to the modulatory role of stress-related brain-gut interactions and its association with psychological factors and emotional state, it proves useful to encourage psychopharmacological treatments and psychosocial therapies, both aiming at reducing stress perception. The aim of this paper is to analyze the effectiveness of psychopharmacological treatment and psychological actions on irritable bowel syndrome.

**Figure 1:**
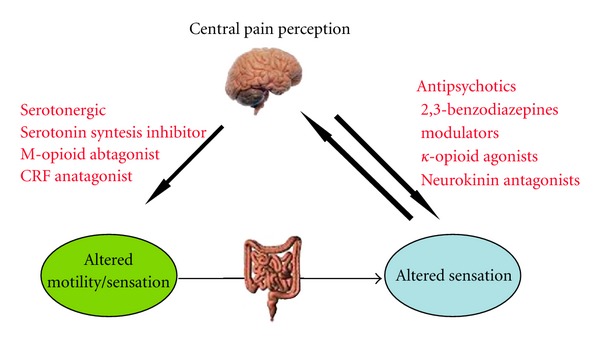
Brain-gut axis and pharmacological approaches for irritable bowel syndrome. This figure shows schematically the targets of the current and novel psychopharmacological therapies for irritable bowel syndrome. The reason to use these approaches is the presence of bidirectional connections between brain and gut: according to this pathophysiological model, emotional and cognitive centers of the brain, particularly those involving pain perception, are linked with peripheral functioning of gastrointestinal tract, and vice versa, thus, conditioning intestinal motility, sensation, and inflammation.

Figure 1 shows schematically the targets of currents and new psychopharmacological therapies for IBS.

## 2. Antidepressants

Among the currently available classes of drugs for the treatment of IBS, antidepressants are useful because of their analgesic properties, independent of their mood-improving effects, and they may therefore be beneficial to patients with neuropathic pain [39–42].

Long-term use of all antidepressants makes it possible to enhance glucocorticoid signaling and to inhibit overactivity of corticotrophin-releasing factors in the brain and presumably in the periphery. Each class of antidepressants affects several transmitters via reciprocal actions between amine and neuropeptide systems and reduces excessive cytokine release associated with various conditions in which inflammatory cytokines play a role [43].

Antidepressants alter receptor sensitivity, which in all cases is believed to result in enhanced serotonin neurotransmission. In particular, tricyclic antidepressants (TCADs) increase the sensitivity of postsynaptic serotonin receptors and downregulate alpha-2 presynaptic receptors and heteroreceptors; their analgesic effects are also mediated by blockage of a class of voltage-dependent sodium channels in extrinsic sensory neurons [44]. They also antagonize muscarinic acetylcholine receptors [45]. These antimuscarinic effects of TCADs are responsible for many of their side effects, including constipation, dry mouth, and blurred vision [46]. However, slowing of GI transit may be of therapeutic advantage in diarrhea-predominant IBS [47]. In contrast, selective serotonin reuptake inhibitors (SSRIs) and serotonin-norepinephrine reuptake inhibitors (SNRIs) reduce the sensitivity of 5HT-1A autoreceptors and heteroceceptors, whose downregulation is believed to play the most important role in the antidepressant, anxiolytic, and analgesic effects of antidepressants. In this connection, serotonin acts as a secretagogue and tends to stimulate GI motility [46]. Thus, SSRIs could be particularly useful in patients with constipation-predominant IBS [6].

A 2009 meta-analysis that included 13 placebo-controlled trials of antidepressants in 789 adults with IBS concluded that antidepressants were significantly more effective than a placebo for the relief of pain and global symptoms (relative risk of IBS symptoms persisting 0.66, 95% CI 0.57 to 0.78) at a duration of therapy ranging from one to three months [47]. The number needed to treat one patient was four. The treatment effects were similar for SSRIs and TCADs. A similar conclusion was reached in a 2009 position statement from the American College of Gastroenterology Task Force on IBS [48].

Three further RCTs have been published since the meta-analysis was published, but the results were conflicting, with two demonstrating a benefit of TCADs and SSRIs [49, 50] and a third demonstrating no benefit of SSRIs [51]. However, when the results of these trials are incorporated into the prior meta-analysis, the benefit of both TCADs and SSRIs remains reassuringly similar [52].

A systematic review performed by the Cochrane Library, and evaluating bulking agents, antispasmodics, and antidepressants for the treatment of IBS (but without considering the safety of these drugs), emphasized that “antidepressants are effective for the treatment of IBS” [53], providing a statistically significant benefit over placebo for abdominal pain, global assessment and IBS-symptoms score. Subgroup analyses for SSRIs and TCADs showed a statistically significant improvement in global assessment for SSRIs and a statistically significant improvement in abdominal pain and symptoms score for TCADs. The authors concluded that antidepressants could be used in patients who seek drug therapy and who have not responded to antispasmodics, but considering that their effectiveness may vary with individual patient features.

With regard to the practical use of antidepressants in IBS, improvement in neuropathic pain with TCADs occurs at lower doses than required for treatment of depression. The initial doses of the drugs should be administered, as a result, at a low dosage, titrating them to pain control and tolerance.

In case of partial response to treatment, it is prudent to increase the dose of the drug, reassessing response and tolerability every 4–6 weeks. With an adequate dosage, some response in the first 6–8 weeks should be apparent, but remission can occur. If there is no response at 6 weeks, and compliance and treatment intensity appear to be adequate, switching to another class may obviate the need for referral for psychiatric consultation. Whether to persist or refer remains a clinical decision between the physician and patient. For some patients, the use of concomitant benzodiazepines for anxiety control may help with compliance and allows more optimal control of symptoms [54].

In contrast, antidepressant efficacy is unproven in children, as illustrated in a multicenter trial of 83 children with functional GI disorders, in which they were randomly assigned to amitriptyline or a placebo for four weeks, considering, as the primary end-point, the child's assessment of pain relief and sense of improvement [55]. At four weeks, there was no significant difference between amitriptyline and the placebo in the frequency of attaining the primary end-point (63 versus 58 percent, *P* = 0.85). The authors also noted that a longer period of treatment and a higher dose of antidepressants may have produced different results and that there may be a large placebo effect in children due to multiple factors; indeed, in another trial of 33 adolescents assigned to amitriptyline or a placebo, the first one was effective in reducing diarrhea and pain after a longer period of treatment [56].

This finding is also emphasized in the review performed by the Cochrane Library, where the authors conclude that clinicians must be aware that the existing randomized controlled evidence is limited to studies on amitriptyline and revealed no statistically significant difference between amitriptyline and a placebo for most efficacy outcomes in children and adolescents. Furthermore, antidepressants can lead to substantial, sometimes life-threatening adverse effects, and consequently, until better evidence evolves, clinicians should weigh up the potential benefits of antidepressants in pediatric patients [57].

Although standard antidepressants of the tricyclic and serotonin reuptake inhibitors classes have been assessed in meta-analyses, as mentioned above, an example of a novel class of centrally acting agents that has not been assessed extensively is the atypical antipsychotic quetiapine, which ameliorates anxiety and sleep disturbances, augments the effect of antidepressants, and provides an independent analgesic effect [58].

The use of atypical antipsychotics could be considered in the setting of patients with severe IBS and coexistent severe psychiatric comorbidity. In this connection, in a study on the medical data of patients with severe refractory GI disorders, Grover et al. [59] reported that, among the 11 of 21 patients still on the medication at followup, 6 demonstrated global relief of symptoms and 9 were satisfied with treatment. The other 10 patients discontinued therapy because of the drug's side effect of somnolence or because of lack of GI efficacy. This is a very specific group of patients with known and significant psychiatric diagnoses. This approach requires further blind studies before it can be endorsed; physicians without experience with this class of agents should probably avoid prescribing them [58]. As a consequence, one should not recommend its use until the evidence yields solid data regarding the efficacy and safety of this class of drugs.

## 3. Benzodiazepines

Benzodiazepines enhance the inhibitory effects of gamma-aminobutyric acid (GABA) via potentiation at the GABA-A receptors, diminish norepinephrine neurotransmission, and antagonize the effects of cholecystokinin in the brain and gut. This results in immediate anxiolytic activity [45].

Patients with prominent anxiety are often intolerant of antidepressants and can be treated with benzodiazepine monotherapy [54].

Benzodiazepines receptors were identified in subcortical and hypothalamic regions and appear important in controlling autonomic functions [53], such as motor and sensory activity of the gut [60]: nevertheless they do not exist in the gut [61]. Furthermore, benzodiazepines may lower pain thresholds by stimulating GABA, thereby decreasing brain serotonin. Animal studies on the R-enantiomer of tofisopam (the nonsedative anxiolytic), dextofisopam, showed encouraging results in reducing colon motility and visceral sensitivity with little effect beneath basal conditions [62].

In a phase IIb study of dextofisopam for 12 weeks in 140 patients with IBS, Leventer et al. observed overall symptom relief (primary end point) in 57% of patients as compared with placebo (43% of patients). Although dextofisopam improved stool consistency in men and women, the recurrence rate was only decreased in females. This occurred within one week. The most common side effects were headache and abdominal pain (in 12% of patients in comparison with 4% in the placebo group) which were comparable to placebo. No benefit on bloating, partial defecation, or hospital anxiety and depression scale scores was observed [63].

The main disadvantage of benzodiazepines is the lack of reliable antidepressant efficacy, even if in open-label studies, alprazolam (2–8 mg/day) improves IBS in patients with panic disorders [64]. In another study, Castedal et al. [65] showed a slight effect of midazolam on small bowel motility using manometry; however, phase III related retroperistalsis did not work.

Anxiolytic agents are of limited usefulness in IBS because of the risk of drug interactions, tolerance, potential abuse, and rebound withdrawal. They may, however, be useful for short-term reduction of acute situational anxiety that may be contributing to symptoms [46].

## 4. Future Psychopharmacological Perspectives

On the basis of the results of the clinical trials confirming the effectiveness of centrally-targeted pharmacological interventions, such as with antidepressants, anxiolytics, or a combination of both groups in the treatment of IBS, many other pharmacological agents with similar anxiolytic and/or antidepressant properties have recently been shown to modulate stress-induced visceral hyperalgesia in animal models [66]. Among these agents, opioidergic agents, cannabinoid receptor 1 (CB1), beta-3 adrenergic and somatostatin receptors agonists, N-methyl-D-aspartate (NMDA), CRF1 and cholecystokinin receptor antagonists give encouraging results.

As visceral hypersensitivity is a proposed etiological factor in IBS, as mentioned above, one way to improve a sufferer's symptom might be to modulate pain receptors in the GI tract. Pregabalin and gabapentin, drugs believed to inhibit pain via the alpha-2-deta-subunits of voltage gated calcium channels, have both been studied in small single-center RCTs [67, 68]. Pregabalin was more effective than a placebo, in terms of increasing the sensory thresholds for perception of rectal distension, desire to defecate, and rectal pain, and also demonstrated a trend towards an improvement in average daily pain scores during 3 weeks of therapy [67]. Patients treated with gabapentin demonstrated significantly increased rectal compliance, as well as higher sensory thresholds for bloating, discomfort, and pain during a 5-day treatment period [68].

The efficacy of agonists to the kappa-opioid receptors has also been studied in IBS. After a 100-mg intravenous infusion of fedotozine, thresholds to first perception of colonic distension and pain were significantly increased compared with placebo [69]. A single dose of asimadoline led to an increased pain threshold to colonic distension and significantly reduced the area under the curve of pain intensity in a crossover RCT conducted in 20 IBS subjects [70]. However, further large RCTs of the latter drug have been disappointing [71, 72]. One RCT demonstrated no difference in achievement of the primary end-point, average reduction in pain severity 2 hours after treatment, between asimadoline and a placebo [72], although in a post-hoc analysis there appeared to be a benefit in those with an alternating bowel habit. The second study, conducted in almost 600 IBS patients, also failed to demonstrate any superiority of the drug over a placebo when the proportion of months with adequate relief of IBS pain or discomfort was the primary outcome (37% with active drug versus 33% with placebo) [71]. In contrast to the study by Szarka and colleagues, when a preplanned subgroup analysis was conducted, the drug appeared significantly more effective than a placebo only in IBS-D patients (47% versus 20%).

On the other hand, solabegron, a beta-3 adrenergic agonist, was proposed as a treatment for IBS, based on the finding of functional beta-3 adrenergic receptors on enteric neurons [73]. However, solabegron failed to show any significant effect on human gastrointestinal or colonic transit [74]. A preliminary report of a clinical trial showed that solabegron, 200 mg b.i.d., led to a statistically significant increase in the proportion of female subjects (*P* = 0.019) and possibly subjects of both genders (*P* = 0.06) achieving adequate relief from IBS-related pain and discomfort, as compared with a placebo. There were also improvements in pain scores and the number of pain-free days but (consistent with transit results) no significant changes in bowel symptoms [75].

NMDA receptors are involved in the induction and maintenance of central sensitization during pain states and may also medicate peripheral sensitization and visceral pain. NMDA receptors are composed of NR1, NR2 (A, B, C and D), and NR3 (A and B) subunits, which determine the functional properties of native NMDA receptors. Among NMDA receptor subtypes, the ones containing the NR2B subunit appear particularly important for nociception, thereby, suggesting that NR2B-selective antagonists may be useful in the treatment of chronic pain syndromes [58, 76].

Dextromethorphan is a noncompetitive NMDA receptor antagonist that is widely used as an antitussive agent. In studies conducted on animals, it has been shown to prevent neuronal damage and modulate pain sensation via noncompetitive antagonism of excitatory amino acids. It has proved useful in the treatment of pain in patients with cancer [77]. Somatic heat hyperalgesia has been reported to be associated with increased intestinal permeability in patients with IBS [78]. A subset of IBS patients, but not controls, showed temporal summation of pain in response to a series of six noxious heat pulses. In this setting, perceived intensity of second pain (wind-up) markedly increases with each successive heat pulse. IBS patients who demonstrated temporal summation of pain received 60 mg of dextromethorphan or 50 mg of benadryl in a randomized, double-blind fashion to block wind-up pain. Temporal summation of pain was blocked more effectively by dextromethorphan, an NMDA receptor antagonist, than by benadryl [79].

Regarding the efficacy of cholecystokinin receptor antagonist, the effect of dexloxiglumide, a CCK-1 receptor antagonist, has been studied in female IBS-C patients [80]. However, the drug had no overall effect on colonic transit time. Despite this, the proportion of patients with satisfactory relief of their IBS symptoms was higher with dexloxiglumide than with a placebo (39% versus 11%), although this difference was not statistically significant.

Similarly, pexacerfont, a CRF-1 receptor antagonist, has been evaluated in women with IBS-D [81]. This drug had no effect on orocecal transit time, stool frequency or consistency, or subjective IBS symptoms, including pain and bloating.

Lastly, a study investigating the effect of a slow-release preparation of octreotide on rectal sensitivity and symptoms in IBS patients, although showing an increased threshold of first rectal sensation and improved stool consistency, highlighted the fact that long-term treatment with this agent had no visceral analgesic effect and failed to improve IBS symptoms [82].

In Tables 1 and 2 are showed, respectively, current and novel psychopharmacological approaches for the treatment of IBS.

## 5. Psychological Interventions

Although the etiology of IBS has not been elucidated completely, it is widely accepted that this entity is multifactorial, with external stress and environmental factors playing some role [83]. Since treatment with diet or pharmacological agents alone has been partially successful, psychological interventions have been instituted with promising efficacy. [84].

A broad range of evidence-based mind-body interventions including psychodynamic therapy, cognitive-behavioral therapy (CBT), hypnotherapy, relaxation exercises, or mindfulness meditation has been shown to amend stress coping strategies, both at a cognitive level (catastrophic or self-defeating thoughts) and at a behavioral level (problem solving, especially interpersonal problems) [85, 86]. The symptomatic improvement appears to result from the modulation of stress response, autonomic nervous system balance restoration, and changes in the brain activation pattern in response to visceral stimuli [87].

Interestingly, the symptom course and relapse pattern after psychotherapy seem to differ from those after drug treatment [88]. In one trial, 101 IBS patients received standard medical therapy with or without psychotherapy administered over a 3-month period [89]. During the 3-month intervention period, the improvement was greater in the psychotherapy group than that in the control group. Subsequently, in a 1-year treatment-free followup, the improvement continued in the psychotherapy group, whereas symptoms recurred in the controls, who returned to their initial state [89].

With regard to different psychotherapy approaches, more than 22 trials have been published [47, 90], substantially demonstrating that psychotherapy not only improves psychological symptoms (as anxiety and depression), but also GI symptoms directly [91]. Many of these studies were, however, not selected for meta-analysis because of specific difficulty in controlling unspecific effects. A true “placebo treatment” for such studies is not available and waiting list controls, a frequently established mode in psychotherapy research, may be inappropriate for meta-analytic purposes.

Considering these difficulties, Ford and coworkers, in their meta-analysis mentioned above, observed that while antidepressants are effective in the treatment of IBS, there are few high-quality studies on the use of psychological therapies in IBS; nevertheless, they showed that a range of different psychological therapies was able to significantly reduce physical symptoms in patients with IBS, with studies on CBT providing the greatest evidence [47].

Similarly, in another systematic review and meta-analysis performed by the Cochrane Library [90], the authors concluded that psychological therapies may be superior to the usual care at the end of treatment, although the clinical significance of this benefit is doubtful, considering that the results of the meta-analysis should be interpreted with caution, due to the low methodological quality of the studies included, variability in outcome definitions and small sample size, which resulted in considerable heterogeneity.

According to the authors, psychological therapies in general are not superior to placebos, and the long-term sustainability of the treatment effects of psychological therapies is questionable [90].

In this situation, self-managed support is challenging. A recent systematic review performed by Dorn about self-management support interventions for IBS, including eleven studies, emphasizes the fact that self-management is an essential component of care for IBS [92]. It requires providers to help their patients understand their conditions, manage their own medications, deal with emotional sequelae, develop problem-solving skills, and learn how to find and utilize resources. However, many studies included in this review were of subpar quality, and most of the interventions did not seem feasible for “real world” clinical practice, suggesting that the key challenge for improving self-management in IBS is to develop practical self-management interventions that can be applied across various clinical settings, and then to test them in well-designed clinical trials [92].

CBT is considered the most well-studied psychological treatment for IBS [93], but one limitation is that CBT is rarely available in routine care of IBS [94]. Several factors contribute to this, for example, the lack of trained therapists, high costs of delivering the treatment, and the practical difficulties for patients of scheduling weekly visits at a clinic. Interestingly, several researchers have conducted studies investigating CBT for IBS where participants had therapist contact via the internet (ICBT), defined as a web-based bibliotherapy with an online therapist contact: ICBT proved to be a promising cost-effective treatment modality for IBS as it can be offered to IBS patients on a much larger scale than conventional psychological treatments [95].

In the setting of CBT, a study performed by Lackner and coworkers sought to determine whether the therapeutic phenomenon of rapid response characterizes patients undergoing CBT for IBS; in this study, performed on 71 patients, 30% of CBT-treated patients achieved RR by week 4 of treatment and 90–95% of patients with rapid response maintained gains at immediate and 3 month followup. The authors concluded that rapid response is a potentially important prognostic outcome indicator that has important implications for developing step care approaches for IBS patients [96].

Another study, performed by Reme and coworkers, wanted to examine predictors of treatment outcome after CBT and antispasmodic treatment for patients with IBS in primary care at 12 months after treatment ended. In this study, lower levels of psychological distress (anxiety and depression) at baseline predicted a good outcome in the mebeverine group, but not in the mebeverine + CBT group; in the adjusted model for the mebeverine + CBT group less adaptive IBS related behavioral coping predicted a good outcome [97].

With regard to hypnosis, an important observation has been made by researchers working on its role in treating IBS [98]. Tan et al. and Wilson et al., respectively, observed in their systematic reviews (in spite of both comprising uncontrolled trials) that hypnotherapy was effective in the management of IBS; however, these authors also recommended better quality trials [99, 100].

A Cochrane review and meta-analysis, including four studies, suggests a beneficial effect of hypnosis in the short term, although this result needs to be interpreted with caution, due to the small size and methodological flaws of the studies included, but emphasizes that hypnotherapy appears to be a safe intervention that could be tried in patients who fail standard medical therapy [101].

Curiously, the IMAGINE study [102] showed that if hypnotherapy is effective and if there is no difference in efficacy between individual and group hypnotherapy, this group form of treatment could be offered to more IBS patients, at lower costs.

Another interesting study performed by Lindfors and coworkers showed how gut-directed hypnotherapy is an effective treatment alternative for patients with refractory IBS, but the effectiveness is lower when therapy is given outside highly specialized research centers [103].

With regard to the evidence about psychodynamic therapy, one central problem of all psychodynamic therapy interventions is their poor controllability, as they are different from CBT trials: they are not made up of identifiable modules that can be controlled, for example, by dismissing them in the control group, and finally it is less well standardized in terms of its performance (duration, setting) [104]. In a recent systematic review, only two large-scale psychodynamic therapy interventions yielded an odds ratio of 2.92 (1.76–4.83) [105].

Similarly, few data are available about meditation and reflexology. In a small pilot study performed on 16 patients on whom a medication program was tested and who were asked to practice it regularly at home, a significant improvement was seen in flatulence, bloating, and diarrhea, and clinical responses were sustained with continued meditation at 1-year followup [106]; however, it is unclear whether these results demonstrate a genuine therapeutic effect or a placebo effect. With regard to reflexology, only a small single-blind trial has been conducted, involving 34 IBS patients, in a primary care setting, randomized either to a reflexology foot massage or to a nonreflexology foot massage control group; no significant difference was seen in bowel symptoms between the groups [107].

To date, it is not yet clear if psychosocial therapies are more effective than psychotropic agents in the management of IBS; although some researchers claim the superiority of psychological treatments over antidepressants in terms of long-term reduction in health-care costs [108], there is a scarcity of studies comparing the two different treatment modalities of IBS.

## 6. Conclusions

IBS is a chronic relapsing condition, sometimes associated with significant disability, and with a considerable financial burden for the health service, due to the consumption of resources including physician time, investigations, and costs of treatment [6]. The presence of clinically significant psychiatric symptoms in patients with IBS is an indication for psychotropic agents, especially when stress reactivity is observed [45]. Indeed, antidepressants are effective treatments in IBS, probably as a result of their antinociceptive effects, although additional effects on GI transit may be contributory [6]. Whether any beneficial effect occurs via the treatment of coexistent depression remains unclear, but there was no correlation between depression scores and improvements in IBS symptoms in the studies identified in the meta-analyses that examined this issue [47], also considering that, in the case of TCADs, the doses employed for treating IBS were generally much lower than those used for the treatment of depression [6].

The paucity of data available on the safety and tolerability of psychotropic agents in IBS limits their usage to second-line therapy, according to current IBS management guidelines [48, 109], thus, confirmation of existing practices with randomized, controlled trials is strongly needed. 

With regard to psychological therapies, it is not yet clear if they are more effective than psychotropic agents in the management of IBS. The present evidence show that psychological therapies may be superior to the usual care at the end of treatment, although the clinical significance of this benefit is doubtful and the long-term sustainability of the effects of these treatments is questionable; further studies should focus on the longer term effects and placebos, following current recommendations for IBS treatment trials.

## Figures and Tables

**Table 1:** Current psychotropic drugs used for treatment of irritable bowel syndrome.

Classes of drugs	Mechanism of action	Therapeutic issues
Antidepressants (SSRIs, TCADs, SNRIs)	Neurotransmitters reuptake inhibitors	Reduction of abdominal pain; limited data about safety and tolerability
Benzodiazepines	Enhancement of GABA inhibitory effect	Limited use; risk of tolerance and rebound withdrawal; lack of reliable antidepressant efficacy
Atypical Antipsychotics (Quetiapine)	Receptorial antagonism with dopaminergic (D2) and serotoninergic (5-HT2) receptors	Possible use only in patients with severe psychiatric comorbidities; lacking data and evidence about efficacy and safety in irritable bowel syndrome

SSRIs: selective serotonin reuptake inhibitors. TCADs: trycyclic antidepresants. SNRIs: serotonin norepinephrine reuptake inhibitors. 5-HT: 5-hydroxytriptamine.

**Table 2:** Novel psychotropic agents used for the treatment of irritable bowel syndrome.

Classes of drugs	Mechanism of action	Therapeutic issues
Corticotrophin releasing factor antagonists	Modulation of corticoid system	Limited use, under investigation
Opioidergic agents	Modulation of visceral nociception	Limited central side effects, good efficacy
Cholecystokinin receptor antagonists	Modulation of cholecystokinin pathways	Limited use, under investigation
Somatostatin analogues	Inhibition of rectal sensitivity	Limited use and efficacy, under investigation
Beta-3 adrenergic agonists	Modulation of beta-3 adrenergic system	Limited efficacy in bowel symptoms
N-methyl-D-aspartate antagonists	Central and peripheral analgesics	Limited efficacy, restricted on abdominal pain
